# Reduction of n-3 PUFAs, specifically DHA and EPA, and enhancement of peroxisomal beta-oxidation in type 2 diabetic rat heart

**DOI:** 10.1186/1475-2840-11-126

**Published:** 2012-10-11

**Authors:** Lianguo Hou, Kaoqi Lian, Min Yao, Yun Shi, Xin Lu, Lijia Fang, Tianbo He, Lingling Jiang

**Affiliations:** 1 Department of Biochemistry and Molecular Biology, The Key Laboratory of Neurobiology and Vascular Biology, China Administration of Education, Hebei Medical University, No. 361 Zhongshan East Road, Shijiazhuang, 050017, China; 2 School of Public Health, Hebei Medical University, No. 361 Zhongshan East Road, Shijiazhuang, 050017, China

**Keywords:** n-3 PUFA, EPA, DHA, T2DM, FAO, Peroxisomal β-oxidation

## Abstract

**Background:**

There is overwhelming evidence that dietary supplementation with n-3 polyunsaturated fatty acids (PUFAs), mainly EPA (C20:5n-3) and DHA (C22:6n-3), has cardiovascular protective effects on patients with type 2 diabetes mellitus (T2DM) but not on healthy people. Because the T2DM heart increases fatty acid oxidation (FAO) to compensate for the diminished utilization of glucose, we hypothesize that T2DM hearts consume more n-3 PUFAs and, therefore, need more n-3 PUFAs. In the present study, we investigated the changes in cardiac n-3 PUFAs and peroxisomal beta-oxidation, which are responsible for the degradation of PUFAs in a high-fat diet (HFD) and low-dose streptozotocin- (STZ) induced type 2 diabetic rat model.

**Methods and results:**

The capillary gas chromatography results showed that all the n-3 (or omega-3) PUFAs, especially DHA (~50%) and EPA (~100%), were significantly decreased, and the n-6/n-3 ratio (~115%) was significantly increased in the hearts of diabetic rats. The activity of peroxisomal beta-oxidation, which is crucial to very-long-chain and unsaturated FA metabolism (including DHA), was significantly elevated in DM hearts. Additionally, the real-time PCR results showed that the mRNA expression of most peroxisomal beta-oxidation key enzymes were up-regulated in T2DM rat hearts, which might contribute to the reduction of n-3 (or omega-3) PUFAs.

**Conclusion:**

In conclusion, our results indicate that T2DM hearts consume more n-3 PUFAs, especially DHA and EPA, due to exaggerated peroxisomal beta-oxidation.

## Background

Type 2 diabetes mellitus (T2DM) is associated with high cardiovascular morbidity and mortality [1]. In patients with diabetes, the fatty acid (FA) supply to the heart increases to compensate for the diminished utilization of glucose as an energy source. Although the dramatic increase in FA influx markedly increases the fatty acid oxidation (FAO) [2], it also leads to elevated FA and subsequent triglyceride (TG) synthesis in the diabetic heart, causing cellular lipotoxicity and the initiation of cardiac dysfunction [3]. Alternatively, the diabetic heart is characterized by significant alterations in the fatty acid composition of heart membranes. It has been shown that the linoleic acid (C18:2n-6) content is increased [4] and the n-3 polyunsaturated fatty acid (PUFA) content is decreased [5] in the cardiac phospholipids of STZ-treated rats and fructose-fed rats.

Fatty acids are degraded by both mitochondrial and peroxisomal β-oxidation. The fatty acids that are shorter than C20 are primarily oxidized in the mitochondria, and the fatty acids that are greater than C22 (very-long-chain fatty acids, VLCFA), including polyunsaturated fatty acids (PUFA, Polyenoic acids with 20–22 carbon atoms and 3–6 double bonds), are primarily oxidized in the peroxisomes [6]. The fatty acid oxidation of cardiac fatty acids is controlled, in part, at the gene regulatory level [7,8]. The nuclear receptor peroxisome proliferator-activated receptor α (PPARα), a ligand-activated transcription factor, has an important role in the transcriptional control of genes involved in cardiac fatty acid oxidation [2]. Long-chain fatty acids and a variety of related compounds serve as PPARα ligands [9]. Some of the genes encoding peroxisomal (acyl-CoA oxidase, ACOX) and mitochondrial (muscle isoform of carnitine palmitoyltransferase-1, mCPT-1) FAO enzymes have been identified as PPARα targets [10]. Animal studies have shown that in the diabetic heart, some of the changes in mitochondrial function and mitochondrial FAO occur via the PPARα gene regulatory pathway [2,11]. While previous work on the fatty acid changes and mitochondrial FAO of the heart has focused on the STZ-induced diabetic rat, it is clear that the n-3 PUFAs, especially eicosapentaenoic acid (EPA; C20:5n-3) and docosahexaenoic acid (DHA; C22:6n-3), are important for heart function. Furthermore, a higher intake of dietary long chain n-3 PUFAs has an overall beneficial effect on heart diseases [12,13]. Recent studies have highlighted the important role of long chain n-3 PUFAs in vivo not only as a composition of biomembrane but also as PPARα ligands that cause subsequent alterations in hormonal signaling [14,15]. According to this hypothesis, the level of EPA and DHA in the heart could provide information on the risk of heart diseases. In this study, we used a high-fat diet plus a low-dose of STZ to induce the type 2 diabetic SD rat, which is known to develop many features reported in patients with uncontrolled type 2 diabetes mellitus [16,17], including hyperglycemia, polyuria, and loss of body weight. We examined the free long chain n-3 PUFA composition, the peroxisomal FAO activity, and the mRNA expression of genes encoding the peroxisomal FAO enzymes in the diabetic heart to evaluate the role of DHA and peroxisomal FAO in type 2 diabetic hearts.

## Methods

### Induction of experimental type 2 diabetes in rats

Type 2 diabetic rats were induced by the combination of a high-fat diet (HFD) and a low-dose STZ as described by Srinivasan [16]. Male Sprague-Dawleys (SD) rats weighing 200–220 g were obtained from the Experimental Animal Center of the Hebei Medical University, Shijiazhuang, Hebei Province, China. After 1 week of acclimation, the rats were randomly divided into a control group and a challenge group, and they were fed a normal pellet diet (NPD) consisting of 10.3% fat, 24.2% protein and 65.5% carbohydrate and a HFD consisting of 59.8% fat (mainly pork), 20.1% protein and 20.1% carbohydrate. After 8 weeks of dietary manipulation, an oral glucose tolerance test (OGTT) was performed as per the literature method to confirm the insulin resistance (IR) of the challenge group rats [17].

Diabetes was induced by a single intraperitoneal injection of streptozotocin (STZ; Alexis, USA; 25 mg/kg i.p. in a 0.1 mol/L citrate buffer, pH 4.5) given to rats with insulin resistance. The NPD rats (CON group) were given via the i.p. route in a citrate buffer vehicle. One week after STZ administration, rats with fasting blood glucose (FBG) ≥11.1 mmol/L in two consecutive analyses were considered to be the type 2 diabetic rat model (DM group). All animals remained on the assigned diet until the terminal experiment.

After 8 weeks of diabetes, the FBG was performed again: CON (5.0 ± 0.4 mmol/L) and DM (12.7 ± 1.9 mmol/L). Then, the rats were anaesthetized by using 20% urethane (1.2 g/kg intraperitoneally), and plasma (8–10 ml per animal) was immediately collected from the femoral artery and processed into serum. After being washed in ice-cold saline solution, the hearts were weighed and frozen in liquid nitrogen, then stored at −80°C. All experimental procedures were performed in accordance with the guidelines established by the Ethics Review Committee for Animal Experimentation (Hebei Medical University, Shijiazhuang, China).

### Determination of total free fatty acids and triglycerides with the kit

The total free fatty acids and triglycerides in the serum and heart were measured with the commercial assay kits (Jiancheng, China) following the manufacturer’s instructions.

### Analysis of serum and cardiac free fatty acids by capillary gas chromatography

The lipids were extracted from the serum and heart according to a modification of the literature procedures [18] in methanol-dichloromethane (3:1, vol/vol). The supernatant was vortex-mixed with natrii sulfas exsiccatus (as a dehydrant). The dehydrated supernatant was obtained by centrifugation, and it was transferred into a screw-capped glass tube for further fatty acid methylation. Fatty acid methyl esters were prepared by procedures similar to the literature method [19] using an acetyl chloride/methanol reagent.

Then, the fatty acid methyl esters solved in normal heptane were separated by capillary GLC (Agilent 7890A) using a 30 m × 0.53 mm × 1.00 μm polyethylene glycol column (DB-WAX, Agilent). Split injection (10:1) and flame-ionization detection (FID) were achieved at 300°C. The initial oven temperature programming was 150°C isothermal for 1 min, increased to 190°C at 10°C/min, held isothermal for 58 min at this temperature, then increased to 230°C at 20°C/min and held isothermal for 15 min at 230°C with a Nitrogen gas flow rate of 80 cm/s as the carrier gas. Fatty acids were identified by comparison with standard mixtures of fatty acid methyl esters (Fatty Acid methyl esters are listed in Table 1; Sigma, USA), and the results were calculated by using the respective standard curve and expressed as μg/g tissue or μg/ml serum.

**Table 1:** Changes of serum and cardiac FFA composition in CON and DM groups (n = 12)

**Fatty acid**	** Serum (μmol/ml)**		** Heart (μmol/g prot)**	
	**CON**	**DM**	**CON**	**DM**
C16:0(palmitic acid)	324 ± 21	453 ± 56 **	1481 ± 108	1593 ± 118
C18:0(octadecanoic acid)	202 ± 22	334 ± 31 **	2193 ± 197	2835 ± 129**
C20:0(arachidic acid)	3.9 ± 0.2	5.1 ± 0.2 *	13.0 ± 1.1	14.4 ± 1.3
C22:0(behenic acid)	4.7 ± 0.4	7.5 ± 0.9 **	21.9 ± 1.5	21.7 ± 2.9
C24:0(lignoceric acid)	9.1 ± 1.7	8.8 ± 0.7	36.4 ± 4.7	26.0 ± 1.6**
C16:1n-7(palmitoleic acid)	13.1 ± 1.8	12.2 ± 3.0	42.6 ± 10.8	41.5 ± 4.3
C18:1n-9(oleic acid, OA)	83.4 ± 23.3	259 ± 69 **	859 ± 113	1994 ± 209**
C18:2n-6(linoleic acid, LA)	255 ± 28	231 ± 20	1473 ± 99	1724 ± 229**
C20:4n-6(arachidonic acid, AA)	227 ± 15	250 ± 19	1330 ± 130	1318 ± 145
C22:4n-6(docosatetraenoic acid)	1.4 ± 1.1	10.0 ± 1.3**	49.5 ± 8.1	127 ± 11**
C18:3n-3(α-linolenic acid, LNA)	7.4 ± 0.7	2.8 ± 0.4 **	20.5 ± 6.5	11.3 ± 2.2**
C20:5n-3(eicosapentaenoic acid, EPA)	10.1 ± 1.1	8.2 ± 0.5 **	10.0 ± 1.4	ND
C22:5n-3(docosapentaenoic acid, DPA)	3.6 ± 1.3	3.5 ± 1.3	81.7 ± 7.5	47.2 ± 6.9**
C22:6n-3(docosahexaenoic acid, DHA)	47.4 ± 2.1	50.6 ± 3.8	613 ± 62	317 ± 61**
n-6/n-3	7.1	7.9	3.9	8.4

Data represent the means ± SD. * P < 0.05 vs. CON; ** P < 0.01 vs. CON. ND: not detected.

### Analysis of cardiac fatty acid β-oxidation activity

Rat heart homogenates (10% w/v) were prepared in a medium containing 0.25-M sucrose, 1-mM EDTANa3, and 3-mM Tris–HCl buffer at a pH of 7.0. The homogenates were centrifuged for 5 min at 1000 g. The resulting supernatants were used for the following activity assays.

### Assay of the total activity of fatty acid β-oxidation by spectrophotometry

Both mitochondrial and peroxisomal β-oxidation pathway contains the reaction of NAD^+^ to NADH + H^+^. Therefore, the total activity of mitochondrial and peroxisomal β-oxidation was routinely assayed by the palmitoyl-CoA-dependent NAD^+^ reduction method [20]. The assay mixture contained a 30 mM potassium phosphate buffer at pH 8.2, 0.5-mM NAD, 0.2-mM CoA, 1-mM dithiothreitol, 0.15 mg/ml of bovine serum albumin, 0.01% Triton X-100, and, at most, 500 μg of protein/ml of assay. The temperature was maintained at 37°C. The reaction was initiated by the addition of 50-μM palmitoyl-CoA. NAD reduction was followed spectrophotometrically at 340 nm.

### Assay of peroxisomal β-oxidation activity by luminometric assay

The heart homogenates were prepared exactly the same as the total activity of fatty acid β-oxidation detection. Only the peroxisomal β-oxidation pathway contains the reaction of H_2_O_2_ production and H_2_O_2_ content can be detected exactly by luminometric reaction. The luminometric assay for peroxisomal β-oxidation detection was performed according to Osmundsen [20]. Briefly, an assay mixture was used that contained 0.1-M potassium phosphate, 20-μM luminol, 4-mM NaN_3_, 10 μg of horseradish peroxidase/ml, 0.5 mg of defatted bovine serum albumin/ml, 25-μM palmitoyl-CoA, 100-μM NAD and 5-mM EDTANa_3_, at pH 8.5.

The reaction was initiated by the addition of 10 μl of heart homogenates. The temperature was maintained at 25°C. After 10 min, the mixture was immediately transferred to a sample chamber of multifunction chemiluminescence detector IFFS-A. Then, the luminol mixture was injected, and the relative luminous intensity at its peak height was recorded. Then, the H_2_O_2_ amount was calculated by using a standard H_2_O_2_ curve. Finally, the activity of peroxisomal β-oxidation (mU/mg) was calculated by using the following formula: 1000 × H_2_O_2_ (μM)/[reaction time (min) × protein concentration (mg/L)].

### Assay of mRNA by quantitative real-time PCR

The total RNA was extracted from the heart using the PROMEGA SV Total RNAs Isolation System (PROMEGA Inc., CA, USA). Both the synthesis of complementary DNA (cDNA) from 2000 ng of total RNA and the subsequent PCR amplification was carried out by the SYBR PrimeScript RT PCR kit (Takara, Tokyo). The amplification and detection were performed with a Rotor Gene real-time detection system 6.1 (QIAGEN, Valencia, CA, USA). Gene-specific primers used in the present study (Table 2) were designed using the sequences accessible in the NCBI Reference Sequence and the software Primer Premier 5.0. To demonstrate the specificity of the PCR product, a melting curve analysis was performed after PCR, and each curve was confirmed to be a single peak (data not shown). The thermal cycling program was as follows: 30 s denaturation step at 95°C followed by 50 cycles of 5 s denaturation at 95°C, 20 s annealing at 60°C, and 20 s extension at 72°C.

**Table 2:** Primers for real-time PCR analysis

**Gene**	**Forward primer**	**Reverse primer**	**Product (bp)**
18S	CGCCGCTAGAGGTGAAATTC	CCAGTCGGCATCGTTTATGG	149
PPARα	TGGAGTCCACGCATGTGAAG	TGTTCCGGTTCTTTTTCTGAATCT	113
m-CPT1	AAACATCACTGCCCAAGC	ACTCCATGCGGAAATAGG	108
ACOX1	GTTGATCACGCACATCTTGGA	TCGTTCAGAATCAAGTTCTCAATTTC	78
ACOX3	TGGAGAAGGAACGAGAACTGAAC	ACATGTGGAGGACACATTTGTTG	165
LBP	TTTTAAAATGGGACCCTTCAGAGT	CCCCTTGCGAATTTTCCAA	70
DBP	GGTGGTAAAGAAAGTAAATG	AATTGTGATGGTCGTGTC	148
THLA	GGTATGAGATCAATGCTGCC	GGTGCATTTGGACATTTGTAG	131
THLB	GGCACAAGGGCATCCAATC	GTGCGCTGTCTTTGGTTCAA	51
SCPx	TATGGAATGTCTGCCTGTCC	CCAGTGCTTCATAAGTCAGG	510

### Statistical analysis

Results were expressed as the mean ± SD. The difference was analyzed by Student’s t test. Correlation was determined with the spearman correlation test (SPSS 13.0). P < 0.05 was considered statistically significant. Data were expressed as the mean ± SD. In the case of quantitative real-time PCR data, Ct values of the test genes were normalized by the average Ct values of 18S rRNA for each pooled sample. Fold changes were obtained using 2 ^(−ΔΔCt)^, and standard deviations of fold changes were determined according to the literature procedures [21].

## Results

### Total free fatty acids and triglycerides in serum and heart

At the end of the experiment, serum and cardiac total free fatty acids (FFA) and triglycerides (TG) were measured in the rats of the two groups. As shown in Table 3, serum FFA levels were 1.69-fold higher (1.56 ± 0.08 μmol/ml, p < 0.01) in the DM rats than in the CON rats (0.58 ± 0.11 μmol/ml), and serum TG levels were nearly 4-fold higher (18.18 ± 4.12 μmol/ml, p < 0.01) in the DM rats than in the CON rats (3.75 ± 1.68 μmol/ml). In comparison with CON (0.29 ± 0.05 and 0.75 ± 0.14 μmol/g prot for FFA levels and TG levels, respectively), the DM rats increased cardiac FFA levels by 0.69-fold (0.49 ± 0.11 μmol/g prot, P < 0.01) and TG levels by 1.45-fold (1.84 ± 0.42 μmol/g prot, P < 0.01).

**Table 3:** At the end of the experiment, serum and heart total FFA and total TG in CON and DM groups (n = 12)

	**Serum (μmol/ml)**		**Heart (μmol/g prot)**	
	**CON**	**DM**	**CON**	**DM**
total FFA	0.58 ± 0.11	1.56 ± 0.08*	0.29 ± 0.05	0.49 ± 0.11*
total TG	3.75 ± 1.68	18.18 ± 4.12*	0.75 ± 0.14	1.84 ± 0.42*

Data represent the means ± SD. * *P* < 0.01 vs. CON. FFA: free fatty acid; TG: triacylglycerol.

### Fatty acid profile in serum and heart

To further investigate the changes of different fatty acids in diabetic serum and hearts, we observed 14 different types of FFA by capillary gas chromatography assay (Table 1).

In serum, 6 of 14 FAs including SFAs, including C16:0 (~40%), C18:0 (~65%), C20:0 (~31%) and C22:0 (~60%), MUFAs, including C18:1n-9 (~211%), and PUFAs, including C22:4n-6 (~614%), were significantly increased in the DM group compared with the control group. Only the LNA (C18:3n-3) and EPA (C20:5n-3) were 62% and 19% lower, respectively, in DM serum than in CON serum. Additionally, there was no significant change in n-6/n-3 ratio between the two groups.

In the heart, 4 of 14 FAs, including SFAs, including C18:0 (~29%), MUFAs, including C18:1n-9 (~132%), and PUFAs, including C18:2n-6 (~17%) and C22:4n-6 (~157%), were significantly increased in the DM group compared with the control group. Although the total FFA of the DM heart was significantly increased, 5 of 14 FAs were significantly decreased. C24:0 was 29% lower in the DM heart than in the CON heart. Most importantly, we found that all the n-3 series PUFAs, including LNA (~45%), EPA (~100%), DPA (~42%) and DHA (~48%), were dramatically decreased in the DM hearts compared to the CON hearts. Moreover, accompanied with a slight increase in the sum of n-6 PUFAs (~11%), the dramatic decrease in sum of n-3 PUFAs made the n–6/n-3 ratio ~115% higher in the DM hearts than in the CON hearts.

### The activity of mitochondrial and peroxisomal β-oxidation in the heart

**Figure 1:**
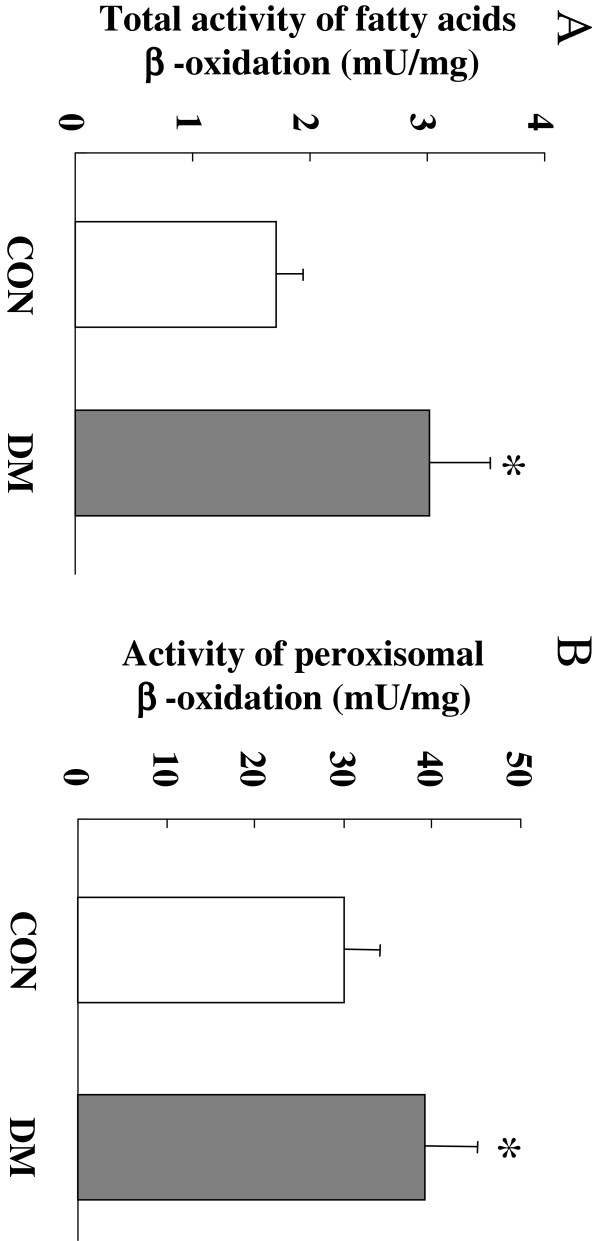
**Comparison of the total activity of fatty acids β-oxidation (A) and the activity of peroxisomal β-oxidation (B) in CON and DM rat hearts (n = 12).** Values are means ± S.E.M. ^**^P < 0.01 vs. CON.

Fatty acids are degraded by β-oxidation. We first analyzed the total β-oxidation (including mitochondrial and peroxisomal β-oxidation) activities in the heart. As shown in Figure 1A, the total fatty acid β-oxidation activities of DM hearts (3.02 ± 0.61 mU/mg prot, P < 0.01) increased by 76% compared with CON hearts (1.71 ± 0.33 mU/mg prot). We further determined the cardiac peroxisomal β-oxidation activity by a luminometric assay. As shown in Figure 1B, the peroxisomal β-oxidation activity in the DM hearts was increased by ~30% (39.2 ± 5.5 mU/mg prot, P < 0.01) compared to the CON hearts (30.1 ± 3.6 mU/mg prot).

**Figure 2:**
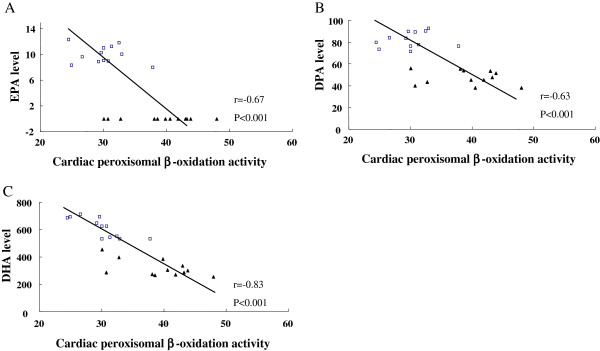
Significant correlation of peroxisomal β-oxidation activity and EPA (A), DPA (B) and DHA (C) levels in rat hearts (□, CON; ▲, DM).

After determining the cardiac fatty acid profile result, we analyzed the relationship of EPA, DPA and DHA content with the peroxisomal β-oxidation activity in heart. A correlation analysis showed that there were negative correlations between the abundance of EPA (r = −0.67, P < 0.001), DPA (r = −0.63, P < 0.001) and DHA (r = −0.83, P < 0.001) and the activity of peroxisomal β-oxidation in rat hearts (Figure 2). Thus, the decreased EPA, DPA and DHA in the DM hearts were thought to be largely attributable to elevated peroxisomal β-oxidation activity.

### mRNA levels of mitochondrial and peroxisomal enzymes

In this study, we obtained the mRNA expression of peroxisome proliferator-activated receptor α (PPARα), muscle carnitine palmitoyltransferase 1 (mCPT-1) and peroxisomal β-oxidation enzyme genes, including acyl-CoA oxidase 1 (ACOX1), acyl-CoA oxidase 3 (ACOX3), D-bifunctional protein (DBP), L-bifunctional protein (LBP), 3-Ketoacyl-CoA thiolase A/B (THLA/B) and sterol carrier protein x (SCPx), in the heart using real-time RT-PCR (corrected by 18S rRNA).

**Figure 3:**
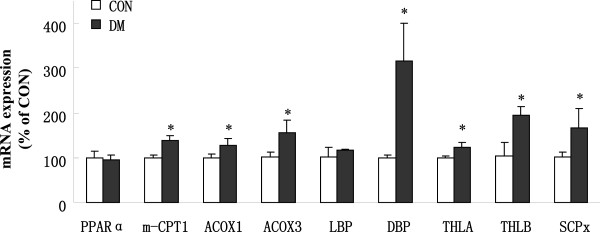
**Change in mRNA expression of PPARα, mCPT-1 and peroxisomal enzymes in CON and DM rat hearts (n = 12 in each group).** Values are means ± S.E.M. ^*^P < 0.01 vs. CON.

PPARα is a critical transcriptional regulator of cardiac lipid and energy metabolism. As shown in Figure 3, the mRNA expression of cardiac PPARα was not changed between the DM and CON group. The mCPT-1 is a key enzyme in mitochondrial β-oxidation in the heart, and the relative expressions of mCPT-1 mRNA in the DM group was higher (P < 0.01) than in the CON group. It indicated that the FA utilization of mitochondria was elevated in the diabetic heart. In peroxisomal pathways of β-oxidation, enzymes of catalytic dehydrogenation are ACOX1 and ACOX3; enzymes of catalytic imbitition and redehydrogenation are LBP and DBP; enzymes of catalytic thioclastic reaction are THLA/B and SCPx [10]. And the expression at the mRNA levels of almost all of the peroxisomal β-oxidation enzymes, except LBP, were significantly enhanced (P < 0.01) in the DM group compared to the CON group. Therefore, the increased peroxisomal β-oxidation activity was caused at least in part by the enhanced mRNA levels of peroxisomal β-oxidation enzymes in the DM heart.

## Discussion

In the heart, FAs contribute approximately 70% of the ATP necessary for normal heart function. During diabetes, there is an increased FA supply to the diabetic heart to compensate for the diminished contribution of glucose as an energy source due to insulin deficiency [22]. Because of the dramatic increase in FA influx compared to FA oxidation, FA and TG accumulation occurs in the heart [3,23], leading to lipotoxicity [24]. Consistent with these reports, we found that although the activity of total and peroxisomal fatty acid β-oxidation was increased, the accumulation of TG and total free fatty acid in the diabetic heart occurred in tandem with increases in serum TG and total free fatty acid.

Mitochondrial β-oxidation constitutes the major process by which most of the short- (<C8 ), medium- (<C14 ), and long-chain (<C22 ) saturated and unsaturated fatty acids are oxidized to generate energy. In the diabetic status, we found a slight increase in the fatty acids of C16:0, C18:0, C20:0, and C22:0 (0.39-fold, 0.65-fold, 0.31-fold, and 0.59-fold, respectively) and a significant increase in the fatty acids of C18:1n-9 and C22:4n-6 (2-fold and 6-fold, respectively). We also found decreases in the fatty acids of C18:2n-6 and C18:3n-3 (0.13-fold and 0.62-fold, respectively) in the serum with an increase in the fatty acids of C18:0, C18:1n-9, and C22:4n-6, (0.29-fold, 1.3-fold, and 1.5-fold, respectively) and a decrease in the fatty acids of C18:3n-3 (0.45-fold) in the heart. Considered together with the increase in total fatty acid oxidation activity and mRNA levels of the mCPT-1 gene, which encodes the rate-limiting enzyme in mitochondrial β-oxidation, it suggests that the type 2 diabetic heart removed more fatty acids from circulation to provide energy though mitochondrial β-oxidation; this supports the common view that in diabetes, the FA supply is in excesses, causing the increased cellular oxidative capacity, intracellular TG and FFA accumulation, which is associated with lipotoxicity [25].

It was reported that in STZ-induced insulin deficiency, because of the decrease in 6-desaturase activity, the key enzyme in the conversion of linoleic acid to long-chain PUFA, the increase in linoleic acid (C18:2n-6) was observed in both cardiac and mitochondrial phospholipids [5]. Because the serum C18:2n-6 was decreased and the cardiac mitochondrial β-oxidation was increased in our results, the increase of cardiac C18:2n-6 may also be due to the decrease of 6-desaturase activity.

Peroxisomes are the major sites of degradation of very long-chain saturated fatty acids and polyunsaturated fatty acids by the sequential action of ACOX1 or ACOX3, DBP or LBP, and THLA/B or SCPx. In the present type 2 diabetic hearts, in addition to mitochondrial oxidation, peroxisomal β-oxidation was also observed to be enhanced; this was indicated by increases in activity and mRNA levels of genes involved in the peroxisomal pathway, together with the decrease in lignoceric acid (C24:0) levels that were β-oxidized only by the peroxisomal pathway [26]. Regarding PUFA, peroxisomal β-oxidation takes a dual function: synthesis and degradation [27,28]. Although peroxisomal β-oxidation participates in the synthesis of DHA in some organs, such as the liver and brain, it has been reported that the rat heart cannot synthesize DHA from circulating ALA because it lacks elongase-2 [29]. Thus, the content of PUFA in the heart depends on the transportation to the heart from circulation and degradation in the heart. In our study, the content of EPA, DPA and DHA were significantly decreased in the DM heart, but changes were slight in diabetic serum compared with CON serum. Moreover, the activity of peroxisomal β-oxidation in the diabetic heart correlated negatively with the content of EPA, DPA and DHA. Therefore, the decrease in cardiac long chain PUFAs, including EPA, DPA and DHA, might be related to the elevated peroxisomal β-oxidation activity observed in this study.

It is worth noting that all the observed n-3 PUFAs were significantly decreased in the type 2 diabetic hearts. Moreover, because of the significant decrease in n-3 PUFAs and the slight increase in n-6 PUFAs, the n-6/n-3 ratio was doubled in the diabetic heart. During the past 3 decades, thousands of epidemiologic, observational, experimental, and randomized controlled studies have been published regarding the cardiovascular protective effects of n-3 PUFAs, especially DHA and EPA. For example, DHA and EPA can ultimately increase arrhythmic thresholds, increase the threshold of ventricular fibrillation, increase heart rate variability, reduce ischemic damage, and favorably affect autonomic tone [30-32]. Furthermore, several authors tended to explain the PUFAs effects in terms of a balance between n-6 and n-3 FAs, rather than the absolute amount of each single molecule. In the most simplistic interpretation, a very high n-6/n-3 ratio is considered detrimental for human health [30]. These data, therefore, showed that the reduction of the n-3 PUFA content (mainly EPA, DPA and DHA) and a high n-6/n-3 ratio might be another factor associated with type 2 diabetic hearts in addition to the accumulation of the ectopic free fatty acids and TG. This may also explain why a higher intake of dietary long chain n-3 PUFA, especially EPA and DHA, is required for heart diseases in type 2 diabetes [12].

In some studies, the activation of PPARα and subsequent enhanced expression of the PPARα target gene, which increases fatty acid oxidation, were observed during insulin resistance and diabetes [33,34]. In our results, although the mRNA expression of PPARα had no changes, the enhanced mRNA expressions of ACOX1, 3 and mCPT-1 (PPARα targets) represented the activation of PPARα in the type 2 diabetic rat heart.

In conclusion, we found that, except for the increasing mitochondrial β-oxidation, the activity of peroxisomal β-oxidation was also significantly increased in type 2 diabetic rat hearts. The increasing activity was, at least partly, due to the elevated expression of genes involved in peroxisomal β-oxidation, especially ACOX1 and 3 (PPARα targets). The enhanced peroxisomal β-oxidation and reduced n-3 PUFA content might be another adverse factor associated with type 2 diabetic hearts.

## Competing interests

The authors declare that they have no competing interests.

## Authors’ contributions

LGH was the principal investigator, involved in designing the study, performing the quantitative real-time PCR assays and writing the manuscript. KQL carried out and analyzed capillary gas chromatography assays. MY and YS helped to perform statistical analyses and write parts of the manuscript. XL, LJF and TBH participated in experiments in vivo and performed the fatty acid beta-oxidation activity assays. LLJ designed and supervised the study as well as helped to draft the manuscript. All authors read and approved the final manuscript.

## References

[B1] Fox CS Coady S Sorlie PD D'Agostino RB Sr Pencina MJ Vasan RS Meigs JB Levy D Savage PJ Increasing cardiovascular disease burden due to diabetes mellitus: the Framingham Heart Study Circulation 2007 115 12 1544 1550 10.1161/CIRCULATIONAHA.106.658948 17353438

[B2] Carley AN Severson DL Fatty acid metabolism is enhanced in type 2 diabetic hearts Biochim Biophys Acta 2005 1734 2 112 126 10.1016/j.bbalip.2005.03.005 15904868

[B3] van de Weijer T Schrauwen-Hinderling VB Schrauwen P Lipotoxicity in type 2 diabetic cardiomyopathy Cardiovasc Res 2011 92 1 10 18 10.1093/cvr/cvr212 21803867

[B4] Hu Q Ishii E Nakagawa Y Differential changes in relative levels of arachidonic acid in major phospholipids from rat tissues during the progression of diabetes J Biochem 1994 115 3 405 408 8056750 10.1093/oxfordjournals.jbchem.a124351

[B5] Ovide-Bordeaux S Grynberg A Docosahexaenoic acid affects insulin deficiency- and insulin resistance-induced alterations in cardiac mitochondria Am J Physiol Regul Integr Comp Physiol 2004 286 3 R519 R527 10.1152/ajpregu.00303.2003 14604840

[B6] Camoes F Bonekamp NA Delille HK Schrader M Organelle dynamics and dysfunction: A closer link between peroxisomes and mitochondria J Inherit Metab Dis 2009 32 2 163 180 10.1007/s10545-008-1018-3 19067229

[B7] Vega RB Huss JM Kelly DP The coactivator PGC-1 cooperates with peroxisome proliferator-activated receptor alpha in transcriptional control of nuclear genes encoding mitochondrial fatty acid oxidation enzymes Mol Cell Biol 2000 20 5 1868 1876 10.1128/MCB.20.5.1868-1876.2000 10669761 PMC85369

[B8] Razny U Kiec-Wilk B Wator L Polus A Dyduch G Solnica B Malecki M Tomaszewska R Cooke JP Dembinska-Kiec A Increased nitric oxide availability attenuates high fat diet metabolic alterations and gene expression associated with insulin resistance Cardiovasc Diabetol 2011 10 68 10.1186/1475-2840-10-68 21781316 PMC3212914

[B9] Koopmans SJ Dekker R Ackermans MT Sauerwein HP Serlie MJ van Beusekom HM van den Heuvel M van der Giessen WJ Dietary saturated fat/cholesterol, but not unsaturated fat or starch, induces C-reactive protein associated early atherosclerosis and ectopic fat deposition in diabetic pigs Cardiovasc Diabetol 2011 10 64 10.1186/1475-2840-10-64 21756316 PMC3143922

[B10] Bilbao E Cajaraville MP Cancio I Cloning and expression pattern of peroxisomal beta-oxidation genes palmitoyl-CoA oxidase, multifunctional protein and 3-ketoacyl-CoA thiolase in mussel Mytilus galloprovincialis and thicklip grey mullet Chelon labrosus Gene 2009 443 1–2 132 142 19465092 10.1016/j.gene.2009.05.008

[B11] Finck BN Lehman JJ Leone TC Welch MJ Bennett MJ Kovacs A Han X Gross RW Kozak R Lopaschuk GD Kelly DP The cardiac phenotype induced by PPARalpha overexpression mimics that caused by diabetes mellitus J Clin Invest 2002 109 1 121 130 11781357 10.1172/JCI14080 PMC150824

[B12] Buse JB Ginsberg HN Bakris GL Clark NG Costa F Eckel R Fonseca V Gerstein HC Grundy S Nesto RW Pignone MP Plutzky J Porte D Redberg R Stitzel KF Stone NJ American Heart Association; American Diabetes Association Primary prevention of cardiovascular diseases in people with diabetes mellitus: a scientific statement from the American Heart Association and the American Diabetes Association Circulation 2007 115 1 114 126 17192512 10.1161/CIRCULATIONAHA.106.179294

[B13] Chicco AJ Sparagna GC McCune SA Johnson CA Murphy RC Bolden DA Rees ML Gardner RT Moore RL Linoleate-rich high-fat diet decreases mortality in hypertensive heart failure rats compared with lard and low-fat diets Hypertension 2008 52 3 549 555 10.1161/HYPERTENSIONAHA.108.114264 18663155 PMC2864132

[B14] Baur LA O'Connor J Pan DA Kriketos AD Storlien LH The fatty acid composition of skeletal muscle membrane phospholipid: its relationship with the type of feeding and plasma glucose levels in young children Metabolism 1998 47 1 106 112 10.1016/S0026-0495(98)90202-5 9440487

[B15] Slee EL McLennan PL Owen AJ Theiss ML Low dietary fish-oil threshold for myocardial membrane n-3 PUFA enrichment independent of n-6 PUFA intake in rats J Lipid Res 2010 51 7 1841 1848 10.1194/jlr.M004069 20219901 PMC2882742

[B16] Srinivasan K Viswanad B Asrat L Kaul CL Ramarao P Combination of high-fat diet-fed and low-dose streptozotocin-treated rat: a model for type 2 diabetes and pharmacological screening Pharmacol Res 2005 52 4 313 320 10.1016/j.phrs.2005.05.004 15979893

[B17] Reed MJ Meszaros K Entes LJ Claypool MD Pinkett JG Gadbois TM Reaven GM A new rat model of type 2 diabetes: the fat-fed, streptozotocin-treated rat Metabolism 2000 49 11 1390 1394 10.1053/meta.2000.17721 11092499

[B18] Coste TC Deumer G Reychler G Lebecque P Wallemacq P Leal T Influence of pancreatic status and sex on polyunsaturated fatty acid profiles in cystic fibrosis Clin Chem 2008 54 2 388 395 10.1373/clinchem.2007.094623 18089657

[B19] Lepage G Roy CC Specific methylation of plasma nonesterified fatty acids in a one-step reaction J Lipid Res 1988 29 2 227 235 3367090

[B20] Osmundsen H Brodal B Hovik R A luminometric assay for peroxisomal beta-oxidation. Effects of fasting and streptozotocin-diabetes on peroxisomal beta-oxidation Biochem J 1989 260 1 215 220 2775184 10.1042/bj2600215 PMC1138648

[B21] Bookout AL Mangelsdorf DJ Quantitative real-time PCR protocol for analysis of nuclear receptor signaling pathways Nucl Recept Signal 2003 1 e012 16604184 10.1621/nrs.01012 PMC1402222

[B22] Dirkx E Schwenk RW Glatz JF Luiken JJ van Eys GJ High fat diet induced diabetic cardiomyopathy Prostaglandins Leukot Essent Fatty Acids 2011 85 5 219 225 10.1016/j.plefa.2011.04.018 21571515

[B23] van der Vusse GJ van Bilsen M Glatz JF Cardiac fatty acid uptake and transport in health and disease Cardiovasc Res 2000 45 2 279 293 10.1016/S0008-6363(99)00263-1 10728348

[B24] Pulinilkunnil T Rodrigues B Cardiac lipoprotein lipase: metabolic basis for diabetic heart disease Cardiovasc Res 2006 69 2 329 340 10.1016/j.cardiores.2005.09.017 16307734

[B25] An D Rodrigues B Role of changes in cardiac metabolism in development of diabetic cardiomyopathy Am J Physiol Heart Circ Physiol 2006 291 4 H1489 H1506 10.1152/ajpheart.00278.2006 16751293

[B26] Poirier Y Antonenkov VD Glumoff T Hiltunen JK Peroxisomal beta-oxidation–a metabolic pathway with multiple functions Biochim Biophys Acta 2006 1763 12 1413 1426 10.1016/j.bbamcr.2006.08.034 17028011

[B27] Ferdinandusse S Denis S Dacremont G Wanders RJ Studies on the metabolic fate of n-3 polyunsaturated fatty acids J Lipid Res 2003 44 10 1992 1997 10.1194/jlr.M300223-JLR200 12897190

[B28] Van Veldhoven PP Biochemistry and genetics of inherited disorders of peroxisomal fatty acid metabolism J Lipid Res 2010 51 10 2863 2895 10.1194/jlr.R005959 20558530 PMC2936746

[B29] Igarashi M Ma K Chang L Bell JM Rapoport SI Rat heart cannot synthesize docosahexaenoic acid from circulating alpha-linolenic acid because it lacks elongase-2 J Lipid Res 2008 49 8 1735 1745 10.1194/jlr.M800093-JLR200 18456640 PMC6075821

[B30] Russo GL Dietary n-6 and n-3 polyunsaturated fatty acids: from biochemistry to clinical implications in cardiovascular prevention Biochem Pharmacol 2009 77 6 937 946 10.1016/j.bcp.2008.10.020 19022225

[B31] Lee JH O'Keefe JH Lavie CJ Marchioli R Harris WS Omega-3 fatty acids for cardioprotection Mayo Clin Proc 2008 83 3 324 332 10.4065/83.3.324 18316000

[B32] Vrablik M Prusikova M Snejdrlova M Zlatohlavek L Omega-3 fatty acids and cardiovascular disease risk: do we understand the relationship? Physiol Res 2009 58 Suppl 1 S19 S26 19857032 10.33549/physiolres.931860

[B33] Buchanan J Mazumder PK Hu P Chakrabarti G Roberts MW Yun UJ Cooksey RC Litwin SE Abel ED Reduced cardiac efficiency and altered substrate metabolism precedes the onset of hyperglycemia and contractile dysfunction in two mouse models of insulin resistance and obesity Endocrinology 2005 146 12 5341 5349 10.1210/en.2005-0938 16141388

[B34] Finck BN Han X Courtois M Aimond F Nerbonne JM Kovacs A Gross RW Kelly DP A critical role for PPARalpha-mediated lipotoxicity in the pathogenesis of diabetic cardiomyopathy: modulation by dietary fat content Proc Natl Acad Sci U S A 2003 100 3 1226 1231 10.1073/pnas.0336724100 12552126 PMC298755

